# Asymmetrical Shift Toward Longer Dry Spells Associated with Warming Temperatures During Russian Summers

**DOI:** 10.1029/2019GL084748

**Published:** 2019-10-30

**Authors:** Hengchun Ye, Eric J. Fetzer

**Affiliations:** ^1^ College of Natural and Social Sciences California State University Los Angeles CA USA; ^2^ Jet Propulsion Laboratory California Institute of Technology Pasadena CA USA

**Keywords:** drought, dry spell duration, climate change, Russia, precipitation, asymmetrical shift

## Abstract

This study examines the changing behavior of summer dry spell duration in response to increasing air temperatures at 517 Russian stations during 1966–2010. We found that the frequency distribution of dry spell duration (as represented by histograms) is becoming skewed toward longer dry spells. This asymmetrical shift is accompanied by mean increases in dry spell duration. This asymmetry is also reflected in exponentially higher increasing rates of dry spell duration toward higher percentiles. Consequently, across Russia, summers have experienced significant increases in 7‐day‐or‐longer dry spells (at 6.1%/°C of warming) and fewer occurrences of 3‐day‐or‐shorter dry spells (at 2.4%/°C). This study suggests that hotter summers favor more frequent prolonged dry spells, exacerbating drought and heat wave conditions during Russian summers as air temperatures continue to rise.

## Key Points

- Change in distribution of summer dry spell duration demonstrated an asymmetrical shift toward longer dry spells as temperatures rose
- Warmer summers led to fewer occurrences of dry spell durations of 3 days or shorter but more occurrences of dry spells of 7 days or longer

## 1 Introduction

Hotter summers over northern Eurasia are generally associated with decreased wet day occurrence and precipitation totals (Ye et al., 2014; Ye et al., 2016) but with higher intensity (Ye et al., 2015; Ye, Fetzer, Behrangi, et al., 2016) and extremes (Ye et al., 2017). Decreased wet day occurrence is likely linked to longer dry spell durations (Fischer et al. 2015; https://iopscience.iop.org/article/10.1088/1748-9326/aaae0d/meta, 2018; Zolina, 2013). Studies of the changing duration of wet and dry spells are a relatively new research area that has only been explored in limited geographical locations. The results of trend analyses of mean dry spell duration appear to vary among geographical regions, seasons, and time periods. For example, opposite trends of mean dry and wet periods are found in different parts of Europe (Kuglitsch et al., 2010; Schmidli & Frei, 2005; Wibig, 2009, Zolina, 2013) and the United States (Groisman et al., 2012; McCabe et al., 2010).

Climate modeling studies suggest that dry spells of longer duration are to be expected in Canada and tropical Africa (Bouagila & Sushama, 2013; Sushama et al., 2010). Regional models projecting fewer summer wet days are consistent with observed increases in multiday dry spells and possibly fewer episodes of short dry spells in Switzerland (Fischer et al., 2014). The Canadian regional climate model projects increases in return levels of maximum dry spell durations over the southern Prairie Provinces during the warm seasons of 2041–2100 if the climate continues to warm (Sushama et al., 2010).

A recent study by https://iopscience.iop.org/article/10.1088/1748-9326/aaae0d/meta (2018) examined the changes in mean and extremes of seasonal wet and dry day duration and suggested that locations with longer dry spells tend to have higher sensitivity to air temperature change and that both wet and dry spells have amplified responses to temperature compared to that of the mean values. To our knowledge, no research has examined the change in symmetry of dry spell duration, which is one of the three main aspects of climate change (Field et al., 2012) and could be accompanied by a shift in the mean (Konapala et al., 2017).

These studies of asymmetry in air temperature change revealed larger increases in higher tail of temperatures based on both long historical records at alpine stations in Switzerland, Germany, and the United Kingdom (Matiu et al., 2016) and on CMIP5 models' outputs (Kodra & Ganguly, 2014).

Studies also have found asymmetrical changes in daily precipitation intensity from both geographical and temporal perspectives (Konapala et al., 2017; Lau et al., 2013; Pendergrass & Knutti, 2018). Since precipitation extreme is the main driver of changes in the mean, more precipitation is falling in fewer but more intense events. The disproportionately larger increases in higher precipitation intensity and extremes (Collins et al., 2013; Lenderink & van Majgaard, 2008; Pendergrass & Knutti, 2018) appear to be consistent with increased convective precipitation occurrence and intensity at the expense of nonconvective storms as air temperature increases (Ye et al., 2016, 2017).

This study specifically focuses on detecting possible changes in symmetry of dry spell duration in relationship to air temperature by examining changes in dry spell length and frequency at 517 Russian stations during the time period of 1966–2010.

## 2 Data and Methodology

Dry spell duration is constructed from daily precipitation records extracted from the Daily Temperature and Precipitation Data for 518 Russian Meteorological Stations (one station has no records and was excluded) available from the Carbon Dioxide Information Analysis Center (Bulygina & Razuvaev, 2012; Ye et al., 2017). This data set includes daily precipitation (total accumulation for each calendar day of 0.1 mm or higher) and minimum, maximum, and mean daily air temperatures. The starting year of 1966 is used due to consistency in rain gauge types, observation practices, and quality control methods for precipitation observation beginning that year (Groisman et al., 1991). As we focus on the paired relationship between dry day duration and air temperature, the number of missing seasons and the length of the time period should not affect our results significantly. Thus, we are able to keep all available stations with varying data gaps as long as each has 25–45 years of available records for the summer season.

Dry spell duration is defined as the total number of consecutive dry days (without any recorded precipitation) between the two nearest dates on which at least 0.1 mm of precipitation was recorded (https://iopscience.iop.org/article/10.1088/1748-9326/aaae0d/meta, 2018). If more than 10% of daily records at a station are missing (for either precipitation or air temperature) for any month of the summer, the season is considered missing for that station. We use two approaches to separate different levels of dry spell duration: (1) length of duration (in days) to examine changes in frequency of each duration level and (2) the percentile values of duration from bottom 20% to top 95% to examine changes in intensity at each individual location.

Seven time series of occurrences of summer dry spell duration of 1, 2, 3, 4, 5, 6, and 7 days or longer are constructed for each station, along with the time series of summer air temperature. Spearman ranked correlation analysis is used to examine the statistical relationships between the occurrence of each dry spell length and summer air temperature. A 95% confidence level is used to define statistical significance. Simple linear regression analysis with occurrence of duration for each dry spell as a dependent variable and air temperature as the independent variable is performed to estimate the rate of change for each degree of air temperature increase.

The time series of dry day duration at corresponding percentile ranks of 20%, 30%, 40%, 50%, 60%, 70%, 80%, 90%, and 95% are extracted from each station. This is done by sorting all dry spell events for each summer. Then, the dry spell duration corresponding to the percentile position is used for each station. The Spearman ranked correlation analysis and linear regression analysis are also applied to each station to reveal relationships between changing dry duration length of each percentile rank and summer mean air temperature, to reveal any differences in dry spell occurrence with temperature.

Finally, the distribution of dry spell duration associated with air temperature is constructed using histograms to visualize the changes between summers of (1) below‐ and above‐mean summer air temperature and (2) the two hottest and coolest summers for each station. The summer temperature is based on each individual station. This way, each station has half of summers above and another half of summers below its mean summer temperature. Thus, in a given year, some stations may have above‐normal temperatures and others may have below‐normal temperatures.

## 3 Results

**Figure 1:**
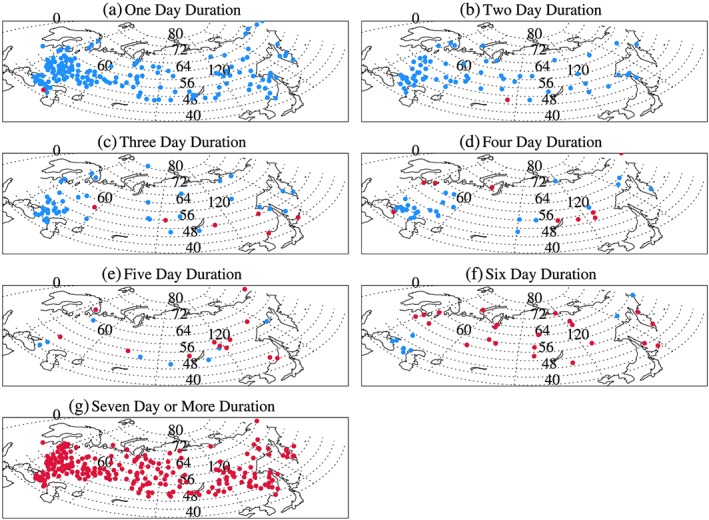
Distribution of statistically significant correlations between the occurrence of various lengths of dry spell and summer air temperature. Red: positive correlation; Blue: negative correlation.

Correlation between the occurrence of dry spells and summer air temperature (Figure 1) shows a dominant negative relationship for 1‐day (164 stations are statistically significant and the largest negative correlation coefficient is −0.59) and 2‐day spells (66 stations are significant). There is a single exception of one station for 1‐day spells located near the west side of the Caspian Sea (Figure 1a) and a single exception for 2‐day spells located in south central Russia (Figure 1b) where statistically significant positive correlation is found. However, relationships for 7‐day‐or‐longer spells are dominantly positive (228 stations are significant and 481 stations have positive correlation with the highest correlation coefficient of 0.65). For 3‐ to 5‐day spells, a small number of stations have statistically significant negative or positive correlations scattered over the study region. For 3‐day spells, significant negative correlations are found over European Russia, northern Siberia, and coastal eastern Siberia and six positive correlation stations are scattered across southern regions (Figure 1c). For 4‐ and 5‐day spells, there are no definite geographical patterns and few statistically significant stations (Figures 1d and 1e). For 6‐day dry spells, 22 stations across northern Eurasia and Siberia show significantly positive correlations with temperature (Figure 1f), while several negatively correlated stations are found over southern European Russia. Dry spells of 7 days or longer have significant positive correlation with temperature everywhere (Figure 1g).

**Figure 2:**
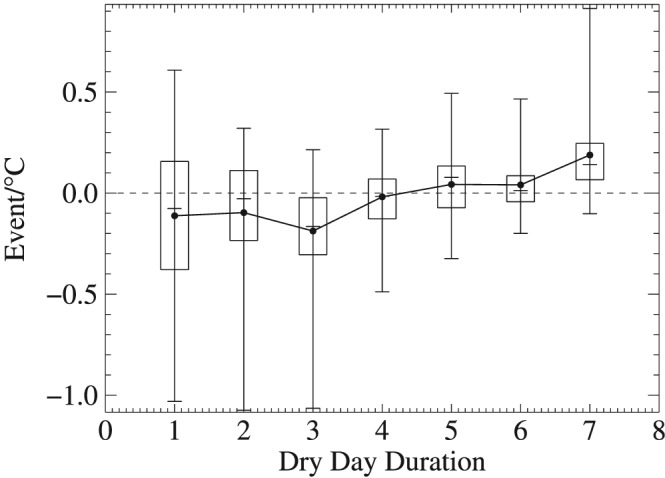
The rate of change for dry spell occurrence of various durations associated with each degree of air temperature increase for all stations. Each box includes 25–75 percentiles of change rates among all stations; short horizontal lines inside boxes are median values for all stations; the dots connected by a line are mean values corresponding to each duration.

The change rate of dry spell event per degree of air temperature increase shows that the majority of negative values are found for dry spells of 3 days or shorter and then switches to positive values for events with 5‐day‐or‐longer duration. The highest positive values occurred for 7‐day‐or‐longer dry spells with the mean and medium rates at 0.19 event/°C and 0.14 event/°C, respectively (Figure 2).

**Figure 3:**
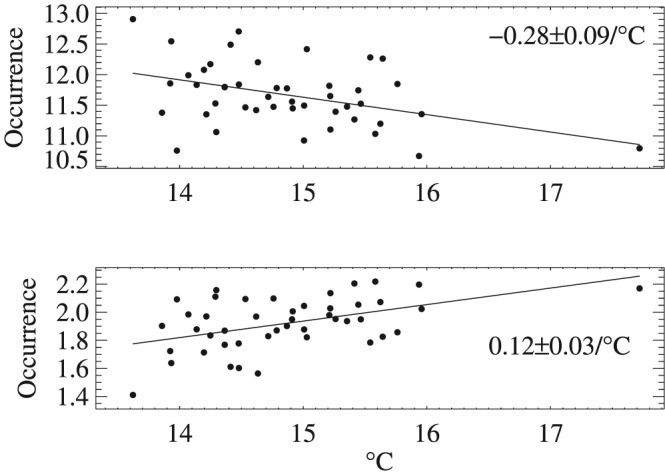
Occurrence of 3‐day‐and‐shorter (upper panel) and 7‐day‐and‐longer dry spells averaged from all available stations corresponding to the average summer seasonal mean air temperature.

Due to the consistency of the relationship among most stations in Figure 1, occurrence of 3‐day‐and‐shorter spells and 7‐day‐and‐longer spells are averaged from all available stations for each summer and plotted against corresponding averaged summer surface air temperatures (Figure 3). A statistically significant increase rate of 0.12 event/°C (or 6.1%) occurs for 7‐day‐and‐longer dry spells and a decrease rate of −0.28 event/°C (or −2.4%) occurs for 3‐day‐and‐shorter dry spells. Both relationships are statistically significant (up to a 99% or higher confidence level). Note that a much higher summer mean temperature occurred in 2010, the last year of the data records. This is the famous July mega‐heat wave in Russia that broke records since 1880 (Dole et al., 2011) and persisted in Eastern Europe into August (Barriopedro et al., 2011). In addition to the significant positive temperature trends among most stations, 2010 has the lowest number of stations reporting data (a total of 335 stations, which is 45 stations fewer than in the next lowest year of 1969). Analyses were also performed without including the summer of 2010 to test the robustness of these results. The mean rate of increase for 7‐day‐or‐longer dry spells is 0.13 events/°C and the decrease rate for 3‐day‐or‐shorter dry spells is −0.27 events/°C, very similar to those that included the 2010 data. There are several reasons that one extreme hot year does not affect our overall conclusion: (1) We are examining the paired relationship between temperature and dry day duration and missing both for some stations should not significantly affect signals. (2) Given the sample size of 25–45, adding or removing one sample does not change the robustness of the relationships.

The summary of correlations results between the individual stations' mean dry spell duration of each percentile and the corresponding summer seasonal mean air temperature is listed in Table 1. It is clear that correlations are dominated by statistically significant positive values, and more stations have significantly positive correlations as the percentile increases.

**Table 1:** Mean Values of Dry Spell Duration by Occurrence Percentile and the Number and Percentage of Stations that Have Statistically Significant Correlations Between the Mean Duration of Each Percentile and the Air Temperature

Percentile	20th	30th	40th	50th	60th	70th	80th	90th	95th
Mean duration (days)	1.02	1.12	1.38	1.84	2.32	2.93	3.95	5.43	6.98
# of positive corr.	43 8.3%	65 12.6%	123 23.8%	183 35.4%	195 37.7%	224 43.3%	263 50.9%	267 51.6%	283 54.7%
# of negative corr.	5 0.9%	1 0.2%	2 0.4%	1 0.2%	0 0%	0 0%	0 0%	0 0%	0 0%

**Figure 4:**
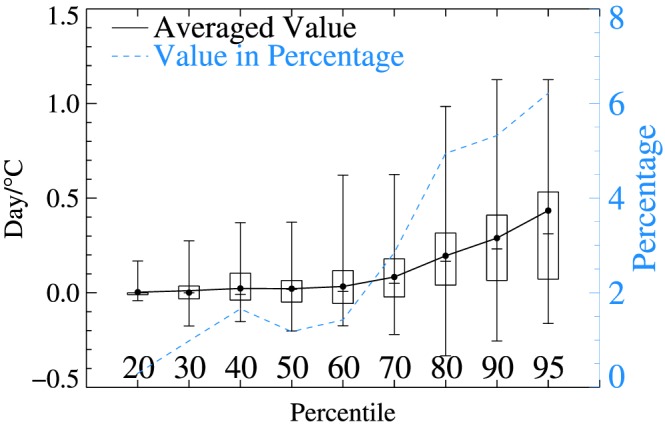
The rate of dry day duration change with air temperature, for each percentile rank for all stations. The vertical line indicates the range of all values and each box includes 50% of stations closest to the median value. The short horizontal line within the box is the median. A solid line connects all mean values and a dashed blue line is the mean value showing the percentage change in dry spell duration.

The mean rates of dry spell duration change with each degree of air temperature increase are positive for all percentile ranks, but the rates increase quickly as the rank of percentile moves up, in an approximately exponential manner (Figure 4). The percentage of change also rises as percentile rank moves up (Figure 4 blue dashed line). It is worth noting that a sharp increase occurred starting with the 70th percentile. This clearly indicates that asymmetrical increases in extreme dry spells are associated with higher air temperatures.

**Figure 5:**
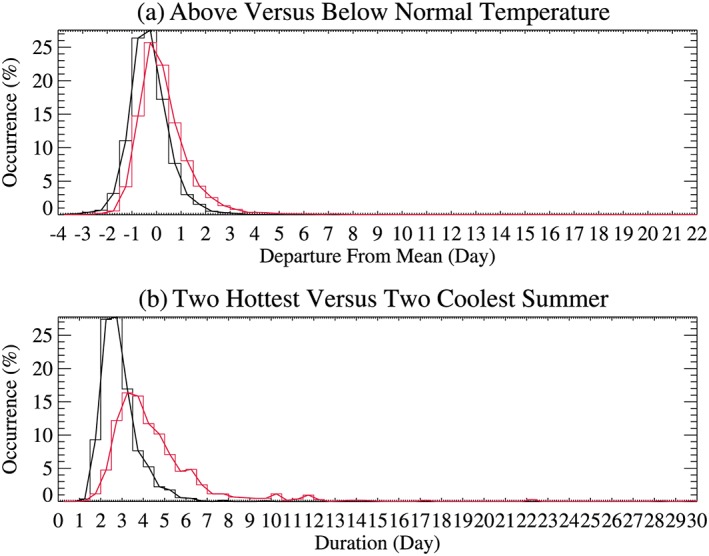
Histogram of occurrence of dry day duration for all stations. (a) Departures for above (red) and below (black) corresponding station's summer climatological mean air temperature and (b) for two hottest (red) versus two coolest summers (black).

For another perspective on associations between the length of dry spells and air temperatures, histograms of dry spell duration departure are constructed for summers when air temperature is above normal (positive departure from the climatological mean of the individual station) and those that are below normal for all stations (Figure 5a). It is clear that dry spell departure shifted toward longer durations for above‐normal temperatures and have a longer histogram tail to the right, indicating longer and more extreme dry spells (a max of 22 days above normal). The longer tail on the long dry spells in combination with little change on the lower tail suggests a case of “Changed Symmetry” in addition to the “Shifted Mean” as defined by the IPCC report (Kodra & Ganguly, 2014). The medium values for dry spell departure are −0.365 and 0.095 days for below‐ and above‐mean summer temperature, respectively, with a shift of 0.46 days.

To examine the extreme summer cases, histograms are constructed for the two warmest versus two coolest summers for each station (Figure 5b). They show more drastic changes in dry spells with temperature, with larger shifts toward longer tails but little change in shorter tails. The medium durations of dry day spells are 2.7 and 3.9 days for the two coolest and hottest summers, respectively, with a shift of 1.2 days.

## 4 Conclusions and Discussion

This study examines structural changes in the occurrence and length of summer dry spells associated with air temperature at 517 Russian stations during 1966–2010. We found that the shift in means toward longer dry day duration associated with higher temperature is mostly due to disproportionate increases in longer dry day duration (higher percentiles). This suggests an asymmetrical shift and increase in variance in which the tail (as represented on a histogram) of dry spell duration widens toward longer durations. When we look at frequency of occurrence, higher summer temperatures are associated with decreased occurrence of short duration dry spells of 3 days or shorter and increased occurrence of long duration dry spells of 7 days or longer across the study region. The mean rate of increase in occurrence of longer dry spells (6.1%/°C) is more than double the mean rate of decrease in shorter dry spells (−2.4%/°C). These results imply that Russia is likely to experience more frequent and longer extreme drought episodes as summer air temperature continues to rise.

Mechanisms underlying droughts and heat waves in Eurasia are reviewed in Schubert et al. (2014). They note that persistent high pressure, and associated blocking of precipitating storm systems, is the dominant cause of Eurasian heat waves. Heat wave conditions are associated with outflow from desert regions to the south of the areas considered in this study, or merging of less frequent northern high pressure systems with those common over southern deserts. The results of this study are consistent with an increased frequency of high pressure systems over Eurasia poleward of about 50 N. Schubert et al. also notes that soil moisture evaporation may act to reduce heat wave length. In this study, heat waves are observed to lengthen, suggesting that evaporative cooling is becoming less effective at reducing temperatures and shortening heat waves.

The results shown here indicate a shift toward longer and warmer dry spells associated with more frequent, persistent high pressure during 1966–2010 over northern Eurasia. Ye et al. (2016a) describes a shift over the same regions and period toward more frequent convective rain and less frequent stratiform rain as temperature and absolute humidity increase, while total rainfall has changed little. The overall picture emerges of longer, warmer dry spells punctuated by less frequent, more intense warm rain events. These conditions broadly describe the climate to the south of the regions considered here. The results of this study and those in Ye et al. (2016a) are consistent with a poleward shift in climate across the breadth of Eurasia during 1966–2010.

The faster increase toward higher percentiles of summer dry spell duration could have contributed to the disastrous 2003 and 2010 mega‐heat waves in Europe and Russia (Barriopedro et al., 2011; Dole et al., 2011). A study over Western Europe found that the length of heat waves has doubled and the frequency of hot days has almost tripled since 1880 (Della‐Marta et al., 2007). Future projections also suggest that mega‐heat waves will be more frequent in much of Europe (Barriopedro et al., 2011).

Heat waves lead to sharp increases in mortality and respiratory emergency visits, as is well documented in many parts of the world, for example, in Spain (Diaz et al., 2018), Germany (Hermann & Sauerborn, 2018), China (Song et al., 2018), and the United States (e.g., Anderson et al., 2018; Vashishtha et al., 2018; Wang et al., 2018). Heat waves also affect agricultural productivity, including hen deaths which reduced egg production in Brazil (Lamarca et al., 2018), increases in female cattle mortality up to 40% in France (Morignat et al., 2018), lowered fertility in dairy cattle (Wolfenson & Roth, 2018), and reduced milk production in the United Kingdom and Australia (Chang‐Fung‐Martel et al., 2017; Fodor et al., 2018). Heat waves also stress ecosystems including temperate coastal Benthic communities (Pansch et al., 2018), affect the sperm quality of birds (Hurley et al., 2018), and induce complex responses of the biosphere (Flach et al., 2018). Prolonged dry spells associated with higher air temperatures that exacerbate drought conditions could have devastating consequences on water supplies, food security, fire hazards, human health, and global politics. Russia's 2010 drought reduced grain harvests by one third and triggered policy changes on grain exports and domestic livestock sizes and impacted the food security of countries that rely on Russian grain supplies (Kramer, 2010; Wegren, 2011). The heat wave in summer 2010 was also responsible for 11,000 deaths, a doubling of the mortality rate in Moscow (ABC News, 2010a) and related wildfires that killed 34 people (ABC News, 2010b). The interactions between high temperatures and air pollution from wildfires contributed to more than 2,000 deaths (Dmitry et al. 2014).

Changes in variability and the shape of heat wave distribution could have more profound impacts on human beings and human society since most kinds of damage are directly linked to extremes rather than mean conditions. One study (Ma et al., 2017) noted the importance of accounting for temporal distributions of extreme temperature events due to the delicate balance between damage and repair periods in organisms. Furthermore, temporal variability could be more sensitive to anthropogenic influences as found in a precipitation intensity study (Konapala et al., 2017). Our study provides a new perspective for understanding the changing characteristics of dry spells, upon which future studies in other parts of the world using observational records and climate model outputs can be based. Future studies are also needed to understand the causes and consequences of these changes as part of intricate feedback processes in the climate system and to better prepare societal resilience to a warming climate.
